# Primary hypothyroidism and isolated ACTH deficiency induced by nivolumab therapy: Case report and review

**DOI:** 10.1097/MD.0000000000008426

**Published:** 2017-11-03

**Authors:** Mei Fang Zeng, Li Chen, Hong Ying Ye, Wei Gong, Li Nuo Zhou, Yi Ming Li, Xiao Long Zhao

**Affiliations:** a Department of Endocrinology, Huashan Hospital North; b Department of Endocrinology, Huashan Hospital, Fudan University, Shanghai, China.

**Keywords:** hypothyroidism, immune checkpoint inhibitors, immunotherapy, isolated corticotrophin deficiency, nivolumab, programmed death receptor-1

## Abstract

**Rationale:**

Nivolumab is a monoclonal IgG antibody blocking programmed death receptor-1 (PD1), leading to restoration of the natural T-cell-mediated immune response against the cancer cells. However, it also causes plenty of autoimmune-related adverse events, which often involves endocrine system.

**Patient concerns:**

A 54-year-old male with renal clear cell carcinoma was treated with nivolumab intravenously. Routine monitoring showed elevated thyroid-stimulating hormone and low free thyroxine after the 6th administration of nivolumab. After the 12th administration, he developed general fatigue, recurrent hypoglycemia, and relative hypotension. Laboratory tests showed low sodium, low morning cortisol without correspondence increase of corticotrophin (ACTH). Other pituitary hormones were normal. MRI showed no space-occupying lesions, but heterogeneous enhancement of the pituitary gland.

**Diagnoses:**

Primary hypothyroidism and isolated ACTH deficiency. The etiologies were assumed to be nivolumab induced autoimmune lymphocytic thyroiditis and hypophysitis, respectively.

**Interventions:**

Hormone replacements with levothyroxine and acetate cortisone were given orally. Nivolumab was adjusted to lower dose and longer interval.

**Outcomes:**

The patient felt good after adequate replacement. Nivolumab was returned to routine dose and interval six months later. And the metastasis was not obviously progressed during this time.

**Lessons:**

The present report provides the first detailed presentation of combined hypothyroidism and isolated ACTH deficiency induced by nivolumab. Adrenal deficiency often develops insidiously. We suggest routine monitoring of fasting blood-glucose, blood pressure and serum sodium as well as thyroid function during nivolumab and other cancer immunotherapies. When unexpected fatigue, hypoglycemia, hypotension or hyponatremia appeared, adrenal deficiency should be taken into consideration.

## 1 Introduction

Programmed death receptor-1 (PD1) is an immune checkpoint expressed on T cells, which plays a role in the inhibition of immune responses against self-antigens under normal physiologic conditions. However, PD1 ligands (PD-L1, PD-L2) are expressed on many types of cancer cells, and resulting in the suppression of antitumor immune response.^[1]^ Nivolumab (Opdivo) is a recently developed monoclonal IgG antibody blocking PD1, leading to restoration of the natural T-cell-mediated immune response against the cancer cells. It has been demonstrated promising responses in patients with a variety of malignancy tumors including renal cell carcinoma.^[2]^ However, it also increases T-cell action to normal tissues, and causes plenty of immune-related adverse events (irAEs), which often involves endocrine organs such as thyroid and pituitary.

Here we describe a patient with renal clear cell carcinoma who developed primary hypothyroidism followed by isolated corticotrophin (ACTH) deficiency during nivolumab therapy.

## 2 Case presentation

A 54-year-old Chinese male was diagnosed as renal clear cell carcinoma of right kidney, pT1bN0M0, stage I, in June 2010, and then underwent excisional surgery. He had a history of type 2 diabetes and began subcutaneously injection of premixed insulin (Novolin 30R) since 2014. The fasting blood glucose was controlled between 5 and 8 mmol/L. Blood pressure was slightly elevated (150/95 mm Hg), but no antihypertensive medication was taken.

Bone (right ilium and left femur) and lung metastasis were developed in September 2015. Targeted therapy with sorafenib (a kinase inhibitor, 0.4 mg twice a day) was administered orally. However, drug resistance was noticed 5 months later. Then a switch to oral axitinib (a tyrosine kinase inhibitor, 5 mg twice a day) began in February 2016, but showed little efficacy. Then a combination therapy of PD1 inhibitor was proposed. Before that, radiotherapy of right ilium lesion was done in March 2016. The intravenous administration of nivolumab (160 mg, about 2 mg/kg) was started every 2 weeks from April 2016 in Hong Kong. The drug effect was obvious for right ilium and lung lesions after 6 administrations, but not as the same effective for left femur lesion. To get further relief, radiotherapy on the left femur lesion was done. Meanwhile, routine monitoring of thyroid function showed elevated thyroid-stimulating hormone (TSH 33.83 mIU/L) and low free thyroxine (<5.15 pmol/L) with markedly increased thyroid peroxidase antibody (TPO Ab) and thyroglobulin antibody. Therefore, primary hypothyroidism was diagnosed, and levothyrocine was taken orally. However, thyroid function was normal 1 month before (Table 1).

**Table 1:**
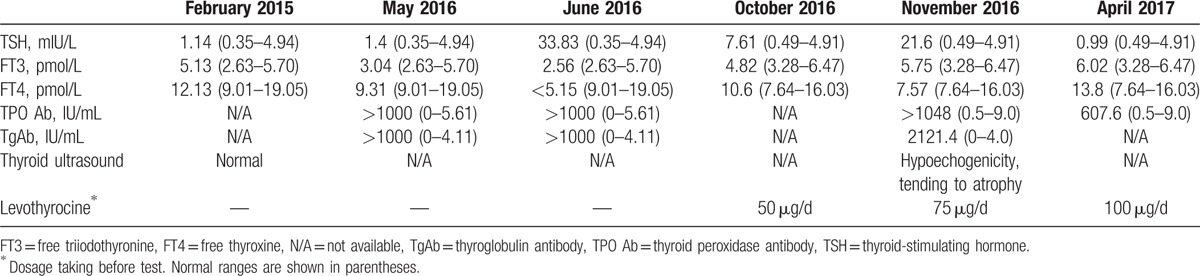
Dynamic changes of thyroid function.

The patient developed general fatigue, recurrent hypoglycemia, and blood pressure dropped to 110/70 mm Hg in October 2016, soon after the 12th administration of nivolumab. He suffered from loss of consciousness at lunch for a while on October 23. Immediately, by being given a sugary drink, he felt better, and was sent to the emergency room. Blood sugar was 6.4 mmol/L; brain computed tomography (CT) scan excluded metastasis. No further tests were taken. He visited our clinic 2 days later, complained of frequent hypoglycemia even without using Novolin 30R. The symptoms suggested hypoadrenalism in our experience. Laboratory examination showed low morning cortisol (3.65 μg/dL) without correspondence increase of corticotrophin (ACTH 33.2 computed tomography pg/mL, reference range 0–46), low sodium (131 mmol/L), normal potassium (4 mmol/L), normal eosinophil (4.2%), and normal white blood cell (6.2 × 10^9^/L). Oral cortisone acetate (12.5 mg once daily) was administered empirically. The patient's symptoms were improved, but suspicious hypoglycemia occurred occasionally. Three weeks later, he was admitted to our hospital for further examination.

On admission, he was doing well physically, his body temperature was 37°C, heart rate was 80 beats per minute, blood pressure was 130/78 mm Hg. His height was 178 cm, body weight was 75 kg, and body mass index was 23.7 kg/m^2^. No pigmentation was noted in his skin or oral mucosa. The pubic and axillary hair was normal. No abnormal findings were elicited by the chest and abdominal physical examination. No edema of lower extremity was found.

**Figure 1:**
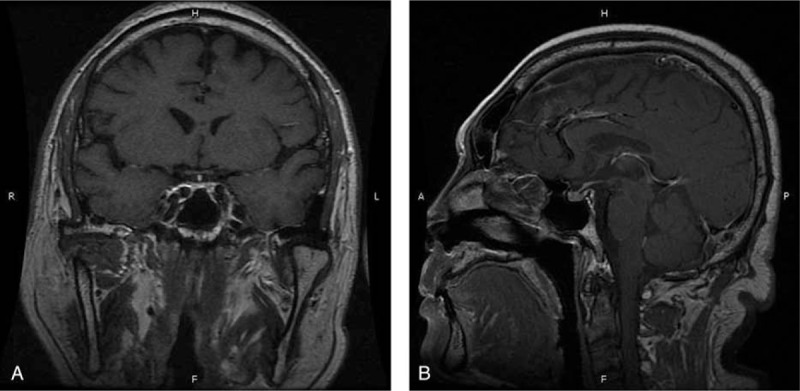
Magnetic resonance imaging (MRI) scan showed heterogenous enhancement of the pituitary gland and enlargement of the basilar part, no space-occupying lesions in the pituitary gland or hypothalamus.

Hormonal examination showed remarkably low early morning plasma ACTH and serum cortisol (see Table 2). Thyroid function showed primary hypothyroidism (Table 1). Other pituitary hormones were normal, shown in Table 2. Diabetes-associated autoimmune antibodies were all negative, including antiglutamic acid decarboxylase (GADA), antityrosine phosphatase antibody, anti-insulin autoantibody (IAA), and islet cell antibody (ICA). No abnormality was found in blood or urine routine test. Magnetic resonance imaging (MRI) scan showed no space-occupying lesions in the pituitary gland or hypothalamus, but heterogeneous enhancement of the pituitary gland (Fig. 1).

**Table 2:**
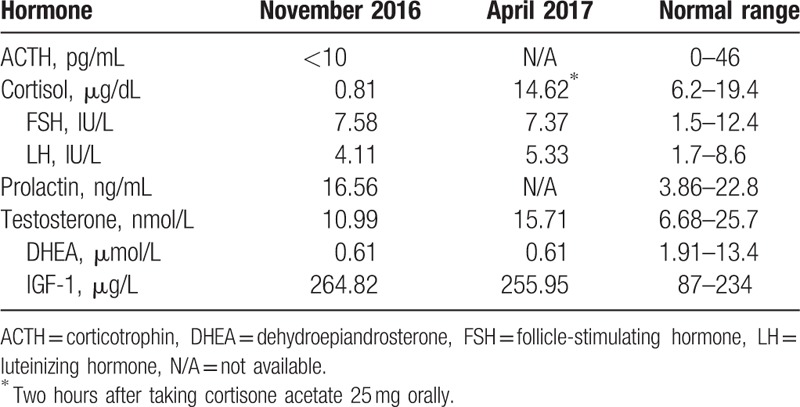
Laboratory findings on admission and follow-up.

New on-set isolated ACTH deficiency was definitively diagnosed on these findings, and the etiology was assumed to be nivolumab-induced hypophysitis. Hormone replacement of oral cortisone acetate was increased to 16.7 mg in the morning, 8.3 mg in the afternoon, and then adjusted to 25 and 12.5 mg, respectively, to achieve a better mental status. Levothyroxine was added to 100 μg once daily finally. During this time, nivolumab was administered with lower dose (1–1.5 mg/kg) and longer interval (every 4 weeks), and the metastasis did not obviously progressed. A follow-up visit showed normal thyroid hormone level in April 2017 (Table 1). Serum cortisol was 14.62 μg/dL at 2 hours after taking cortisone acetate 25 mg orally (Table 2). Also, the patient felt good. After that, nivolumab was returned to routine dose and interval successfully.

## 3 Discussion

Primary hypothyroidism is a common disease, and mostly caused by autoimmune thyroiditis in the area of iodine sufficiency.^[3]^ By contrast, maturity-onset isolated ACTH deficiency is a rare disorder. It is characterized by secondary adrenal insufficiency, normal secretion of pituitary hormones other than ACTH, and absence of pituitary structure defects.^[4]^ In comparison with standard cancer therapy, PD1 inhibitors significantly increased the risk of hypothyroidism, but not for hypophysitis.^[5,6]^ Clinical trials showed that hypothyroidism was the most frequent adverse event, which occurred in 9% (171/1994) of the patients who received nivolumab as a single agent, whereas hypophysitis only occurred in 0.6% (12/1994).^[7]^ Patients who suffered from both thyroiditis and hypophysitis were even rare. The only reported case was about an old Japanese male with malignant melanoma, who exhibited hypopituitarism with slightly hypothyroidism after atrioventricular block.^[8]^ However, there was uncertainty about hypothyroidism, because hypoadrenalism could cause TSH elevated itself, and levothyroxine replacement was not needed.^[9]^

In our case, nivolumab was efficacious, but caused primary hypothyroidism 2 months later, and then induced isolated ACTH deficiency after another 4 months. Before anti-PD1 therapy, the function and ultrasonography of thyroid was normal. Therefore, the abnormal change of thyroid was thought to be autoimmune lymphocytic thyroiditis induced by nivolumab. Unlike classical goitrous Hashimoto thyroiditis,^[10]^ his thyroid was tending to atrophy, and the thyroid function was destroyed quickly as the dose of levothyroxine increased fast. It is likely that the thyroid had been rapidly destructed. Isolated ACTH deficiency was confirmed by hormone evaluation and pituitary MRI test. The etiology of isolated ACTH deficiency is most often hypothesized as lymphocytic hypophysitis or traumatic injury in adults.^[4,11]^ Because our patient had no history of head trauma, combining the enhanced T cell action in anti-PD1 therapy, so that autoimmune hypophysitis was considered to be the etiology. The order of hormone deficiency specific to hypophysitis is as follows: ACTH > TSH > luteinizing hormone/follicle-stimulating hormone > prolactin > growth hormone.^[11]^ Until now, all reported cases of nivolumab-induced hypophysitis were focused on isolated ACTH deficiency.^[8,12–15]^ It is likely that corticotrophin cells are more vulnerable to nivolumab induced autoimmune activation. The precise mechanisms of nivolumab-triggered hypophysitis and thyroiditis remain unclear. A recent study verified that normal thyroid tissue expresses PD-L1 and PD-L2 mRNA and proteins.^[16]^ More studies are needed.

Hypophysitis is usually associated with other autoimmune diseases and most likely comes together with thyroiditis in general population.^[17,18]^ Thyroid autoantibodies were present in 106 out of 151 patients with hypophysitis, as reported in one study.^[17]^ There are no data that provide the prevalence in patients with nivolumab treatment. But it should take into consideration that thyroid hormone replacement could accelerate cortisol clearance and precipitate adrenal crisis in the underlying hypoadrenalism patients.^[19]^ Physicians should pay attention to adrenal function before levothyroxine replacement.

Treatment with high-dose steroids is not needed for nivolumab-related thyroiditis, nor always imperative in nivolumab-induced hypophysitis. Only 2 of 8 (25%) patients received high-dose corticosteroids in previous case reports,^[8,12–15]^ and the proportion was 56% in clinical trials.^[7]^ Others were given hormone replacement therapy directly, so did our patient. High-dose steroids should be used for those patients with adrenal crisis, critical illness, significant hyponatremia, refractory hypotension, or severe headache.

Isolated ACTH deficiency often develops insidiously. Undiagnosed hypoadrenalism can be fatal without timely treatment. Hypoglycemia, hypotension, and hyponatremia were valuable clues for it. We suggested monitoring fasting blood glucose, blood pressure, and serum sodium in patients receiving PD1 antibodies. Moreover, hypophysitis is more common in the treatment of another immune checkpoint blockade, cytotoxic T-lymphocyte antigen 4 antibodies (CTLA4 antibodies, such as ipilimumab), which was reported an incidence of 9.1%.^[20]^ Routine monitoring is also necessary in those patients. The patients should be informed of the common symptoms of adrenal deficiency either.

Usually nivolumab is suggested to be withheld when ACTH deficiency occurs. We also advised the patient to stop nivolumab, but he and his wife were concerned about tumor regrowth and decided to take a reduced dose. They had seen similar measures used by other patients in a nivolumab-using communication “wechat” group. It is showed that lower dose and longer interval of nivolumab can be an alternative option for the patients with hypophysitis. It may achieve better tumor control than withholding dose. However, it need further study to demonstrate which way is more beneficial for patients.

## 4 Conclusions

We report a Chinese patient receiving nivolumab for renal clear cell carcinoma that ultimately experienced thyroiditis and hypophysitis, simultaneously leading to hypothyroidism and isolated ACTH deficiency, respectively. Adrenal deficiency often develops insidiously, so that we suggest monitoring fasting blood glucose, blood pressure, and serum sodium, and also thyroid function in the patients receiving anti-PD1 and anti-CTLA4 therapies. When unexpected fatigue, hypoglycemia, hypotension, or hyponatremia appeared, adrenal deficiency should be taken into consideration. Also, adrenal function should be paid attention to before levothyroxine replacement.
