# Primary hypothyroidism and isolated ACTH deficiency induced by nivolumab therapy: Case report and review: Erratum

**DOI:** 10.1097/MD.0000000000010159

**Published:** 2018-03-16

In the article, “Primary hypothyroidism and isolated ACTH deficiency induced by nivolumab therapy: Case report and review”,^[1]^ which appeared in Volume 96, Issue 44 of *Medicine*, Dr. Li Li Chen's name appeared incorrectly as Li Chen.
